# Palisaded Encapsulated (Solitary Circumscribed) Neuroma Affecting the Lip Aesthetics: Case Report and Literature Review

**DOI:** 10.1155/crid/9705734

**Published:** 2025-06-12

**Authors:** Kaique Alberto Preto, Gabriela Lopes-Santos, Cléverson Teixeira Soares, Ana Paula Moura Martins Delazari, Denise Tostes Oliveira

**Affiliations:** ^1^Department of Surgery, Stomatology, Pathology and Radiology, Area of Pathology, Bauru School of Dentistry-University of São Paulo, Bauru, São Paulo, Brazil; ^2^Department of Pathology, Institute Lauro de Souza Lima, Bauru, São Paulo, Brazil; ^3^Department of Surgery, Private Dental Clinic, Bauru, São Paulo, Brazil

**Keywords:** nerve sheath tumors, palisaded encapsulated neuroma, solitary circumscribed neuroma

## Abstract

Palisaded encapsulated (solitary circumscribed) neuroma (PEN) is a benign nerve sheath tumor, considered a reactive rather than a neoplastic lesion, predominantly found in the head and neck skin and oral mucosa. In the lip regions, less than 20 cases of PEN have been reported, and the differential diagnosis is a challenge. We present a 40-year-old male with a long-standing asymptomatic nodule on the lower lip, causing significant aesthetic discomfort, and a literature review of this tumor in the lips. After surgical excision, the histopathological analysis associated with immunohistochemical markers was essential for establishing the PEN diagnosis in the lower lip. Although rare, the PEN in the lip region can cause significant patient aesthetic discomfort, and histopathological analysis is crucial to distinguish this benign neural tumor from other long-standing lesions in the vermillion of the lips.

## 1. Introduction

Palisaded encapsulated neuroma (PEN), also known as solitary circumscribed neuroma, is a benign nerve sheath tumor typically found in the skin of the head and neck region and the oral mucosa, affecting the tongue, palate, and lips [1–4]. Its etiology remains uncertain, and it was proposed that oral PEN is not a true neoplasm but rather a reactive lesion produced in response to trauma [2].

Despite the favorable prognosis of PEN treated by surgical excision with a very low recurrence rate, misdiagnosis can lead to unnecessary interventions or unjustified alarm, negatively impacting the patient's psychological well-being. Additionally, when located in aesthetic areas such as the lips, PEN can compromise self-esteem and quality of life; therefore, an accurate diagnosis is important.

Histologically, the tumor consists of proliferating spindle-shaped Schwann cells with wavy nuclei, forming fascicles within peripheral nerves. These are accompanied by a partially hyalinized fibrous stroma, with occasional palisading of Schwann cell nuclei [1–5].

In the lip region, less than 20 cases of PEN have been reported, making the differential diagnosis challenging. Here, we describe a case of lip PEN causing significant aesthetic discomfort to the patient and provide a literature review of this tumor in the lips.

## 2. Case Report

**Figure 1:**
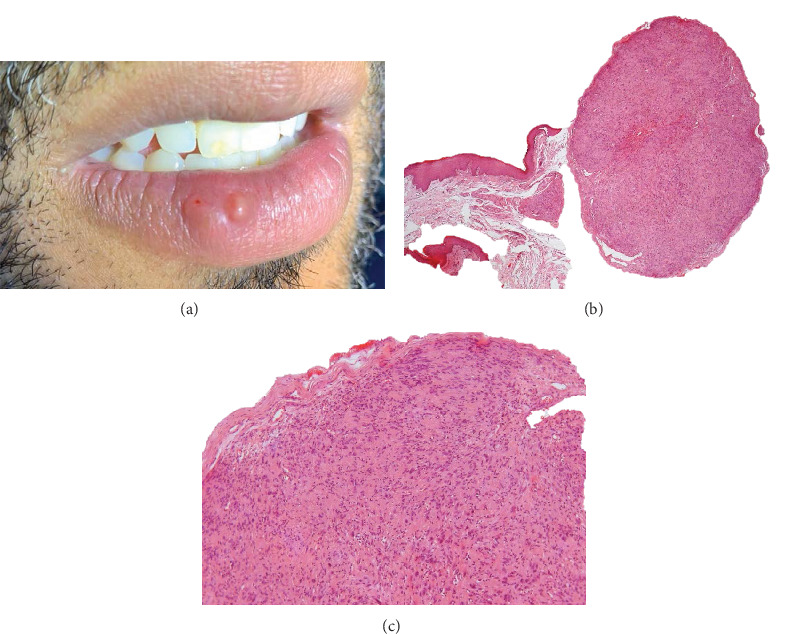
Clinical features of the palisaded encapsulated neuroma (PEN). (a) Presence of well-defined and slightly yellowish nodule on the vermilion of the lower lip. (b) Histopathology showed a well-circumscribed nodule in the submucosa region. (c) Details of the nodule with dense proliferation of spindle-shaped and ovoid cells suggestive of Schwann cells, without cellular atypia, covered by a fibrous capsule (hematoxylin and eosin, original magnification: *B* = 25× and *C* = 100×).

A 40-year-old male presented with a complaint of a “small bump on the lower lip” compromising his aesthetics. The patient had no history of lower lip trauma or relevant medical or family history. On clinical examination, a well-defined, slightly yellowish, sessile-based nodule with a smooth surface, regular contour, and elastic consistency was detected in the vermilion of the lower lip, around 1 cm large. The nodule was asymptomatic but caused aesthetic discomfort and had been noticed for over 30 years (Figure 1a). The clinical diagnosis was a benign salivary gland tumor. An excisional biopsy was performed, and the surgical specimen was sent for histopathological analysis.

**Figure 2:**
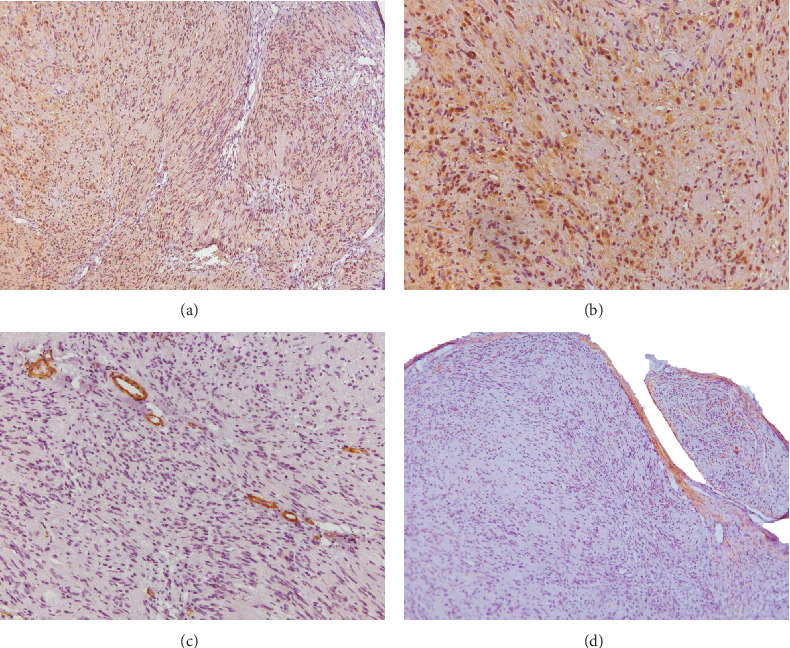
(a, b) The histopathology showed strong immunopositivity for S-100 protein in the spindle-shaped cells, and (c) moderate immunopositivity for alpha-smooth muscle actin (*α*-SMA) was observed in the walls of small blood vessels distributed in the tumor. (d) The focal positivity for epithelial membrane antigen (EMA) was observed in the fibrous connective tissue capsule suggesting the presence of perineurium. Immunohistochemistry, original magnification: A and B (S-100: *A* = 100× and *B* = 200×), C (*α*‐smooth muscle actin = 200×), and D (epithelial membrane antigen = 100×).

The histopathological sections stained with H&E showed, in the submucosa, a well-defined nodule covered by a fibrous capsule (Figure 1b,c). In the nodule, a dense proliferation of spindle-shaped and ovoid cells, mostly with an irregular arrangement or forming fascicles, suggested a bundle-like proliferation of Schwann cells (Figure 1c). Cellular atypia, atypical mitosis, and Verocay body formation were not observed. Immunohistochemical staining revealed strong positivity for S-100 protein in the spindle-shaped cells (Figure 2a,b) and moderate positive staining for alpha-smooth muscle actin (*α*-SMA) located in the wall of small blood vessels (Figure 2c). Epithelial membrane antigen (EMA) positivity was, focally observed in the fibrous connective tissue capsule of the tumor, suggesting the presence of a perineurium (Figure 2d). Additionally, small well-defined neural bundles were detected. The diagnosis was PEN.

After a 1-year follow-up, there was no recurrence, and the patient's aesthetic complaint was resolved.

## 3. Discussion

Lip symmetry seems to be an important aspect of facial beauty, and congenital deformities, tumors, or traumas can impair patients' normal appearance causing significant aesthetic alterations [6]. In the reported case, the patient decided to remove the lesion after 30 years to minimize his aesthetic discomfort.

There are diverse benign and malignant lesions that can occur in the vermilion of the lips, such as salivary gland cysts and tumors, vascular malformations, peripheral nerve sheath tumors, infectious diseases, and squamous cell carcinoma, and the clinical diagnosis can be challenging [3, 4]. Around 10%–16% of nerve sheath tumors in the oral cavity affect the lips [5]. Thus, histopathological analysis is essential for the precise diagnosis of the lesion that manifests in lip regions.

The benign neural tumors such as PEN are uncommon in the vermilion of the lips. Only 15 well-documented cases were found in our literature review using major databases (Scopus, Cochrane Library, EMBASE, BIREME, SciELO, and Web of Science) and gray literature (Google Scholar), as illustrated in Table 1 [6–15]. The PEN affects mainly adult men (58.3%) with an age between the third and fourth decades (mean age of 39.08 years), confirming the features observed in our case report. Nevertheless, unlike the PEN in the lower lip described, this tumor seems to have a predilection for the upper lip (72.7%) (Table 1).

Clinically, PEN in the lip regions can present as either a more superficial or deeper submucosal asymptomatic nodule with different coloration, sizes, and aspects [8–10, 14–17]. In the present case, the clinical diagnosis was a salivary gland tumor, and the hypothesis of traumatic lesions such as mucocele and neuroma was ruled out due to the long duration of the lesion in the lower lip without association with local trauma. Most PEN cases in the lips are treated by surgical excision without recurrence reports [7–17], and in our patient, no signs of recurrence after 1 year of follow-up were detected, and the patient's aesthetic concern was resolved.

The clinical differential diagnosis of PEN on the lips includes several lesions with similar presentations, such as mucocele, a common lesion caused by rupture or obstruction of minor salivary glands, typically appearing as a translucent or bluish nodular lesion often linked to trauma or habitual biting [8, 9]. Schwannoma, a benign nerve sheath tumor, can also present as a firm, painless, slow-growing nodular mass, though it is less common in the lips. Other benign and malignant lesions, such as traumatic fibromas, lipomas, viral papillomas, and squamous cell carcinomas, should also be considered due to their variable clinical appearance in the lips [8–10].

In our case, the lesion affected the patient's aesthetics, but he delayed seeking medical attention due to financial concerns. During this period, he reported feeling significant insecurity and discomfort, as the lesion on his lip was frequently mistaken for contagious lesions, such as herpes simplex. After the lesion removal, the patient reported a substantial aesthetic improvement and an enhancement in his self-esteem.

Histopathological analysis is required to establish the diagnosis of PEN. Typical morphological features of benign neural tumors [2, 3] were found in the present case reported as a well-defined nodule characterized by a proliferation of Schwann cells intensely positive for S-100 protein and covered by a fibrous capsule presenting some immunopositive cells for EMA suggesting perineurium, as illustrated in Figure 1. According to the latest edition of the World Health Organization Classification of Tumors, a histopathological analysis combined with immunohistochemical findings is sufficient for an accurate diagnosis of PEN [2].

Peripheral nerve tumors such as schwannoma, neurofibroma, traumatic neuroma, and perineurioma share a common origin and some histopathological features with PEN [2–4]. Thus, the immunohistochemical markers of benign neural tumors (Table 1), associated with morphological patterns, are crucial to confirm or exclude the diagnosis [3–5, 7]. Microscopically, lip PEN can usually be distinguished from schwannomas without difficulty. The spindle-shaped cells of schwannoma are arranged in two distinct patterns, Antoni A and B, in variable proportions, with few or no intralesional axons, forming completely encapsulated lesions [2–4, 9]. Nevertheless, the morphological distinction between neurofibroma and PEN can be challenging, especially with incisional biopsies, and microscopically, the absence of a fibrous capsule and the disordered distribution of Schwann cells, perineurial cells, and fibroblasts are the main differential aspects observed in neurofibroma compared to lip PEN [3, 4]. In contrast with PEN, perineurioma exhibits a distinctive pattern of concentric arrangement around nerve fibers, with positivity for EMA and negativity for S-100 [3]. Additionally, traumatic neuroma is characterized by morphological disorganization, the absence of a capsule, and often presents inflammatory cells and regenerative nerve fibers [3, 4]. These microscopic differences are crucial for distinguishing perineurioma, PEN, and traumatic neuroma.

Many reported cases of PEN were misdiagnosed due to significant histopathological overlap and difficulty in identifying subtle distinguishing features or potential causal factors [4]. These lesions may instead represent other peripheral nerve sheath or neural origin tumors. This diagnostic challenge, compounded by overlapping histological findings, contributes to the uncertainty surrounding the true incidence of oral PEN [4, 5]. Tumors like schwannomas, neurofibromas, traumatic neuromas, and perineuriomas share a common origin and exhibit considerable histological similarities, complicating accurate diagnosis [4].

This case report reinforces that the PEN in the lip region is a rare benign neural tumor that can cause significant aesthetic discomfort impacting the patient's quality of life. Nevertheless, the histopathological analysis associated with immunohistochemical markers permits distinguishing the PEN from other long-standing lesions occurring in the vermillion of the lip.

## Figures and Tables

**Table 1:** Published clinical and immunohistochemistry data of palisaded encapsulated neuroma located in the lips.

**Authors, year**	**Age**	**Sex**	**Location**	**Immunohistochemistry**
Magnusson, 1996 [7]	23 22 82 26	Male Male Male Female	Upper lip Upper lip Upper lip Upper lip	S-100: +++, EMA: +, and neurofilament: +

Cho et al., 2006 [8]	44	Male	Lower lip	S-100: +++, EMA: +++, and neurofilament: +

Kuyama et al., 2012 [9]	41	Female	Upper lip	S-100: +++, vimentin: +, *α*-actin: (−), and GFAP: (−)

Panthula, 2015 [10]	26	Male	Upper lip	S-100: +++

Ito et al., 2016 [11]	50	Female	Lower lip	S-100: +++, EMA: +++, and neurofilament: +

Cunha et al., 2018 [12]	21	Female	Lower lip	S-100: +++, GFAP: (−), and *α*-smooth muscle actin: +

Leblebici et al., 2019 [13]	45 38 51	Male Male Female	Upper lip Upper lip Upper lip	S-100: +++, EMA: +++, claudin-1: +++, neurofilament: +, and CD34: +++

Jin et al., 2021 [14]	23	Female	Upper lip	S-100: +++

Seol et al., 2022 [15]	63	Female	Lower lip	S-100: +++ and neurofilament: +

Shima et al., 2023 [16]	73	Male	Upper lip	S-100: +++, EMA: +++, and neurofilament: +

Present case, 2024	40	Male	Lower lip	S-100: +++, EMA: ++, and *α*-smooth muscle actin: +++

*Note:* Immunopositivity: intense (+++), moderate (++), low (+), and negative (−).

## Data Availability

The data that support the findings of this study are available on request from the corresponding author. The data are not publicly available due to privacy or ethical restrictions.
